# Predictive multi-imaging biomarkers relevant for visual acuity in idiopathic macular telangiectasis type 1

**DOI:** 10.1186/s12886-018-0737-y

**Published:** 2018-03-05

**Authors:** Jingli Guo, WenYi Tang, Xiaofeng Ye, Haixiang Wu, Gezhi Xu, Wei Liu, Yongjin Zhang

**Affiliations:** Department of Ophthalmology, Eye and ENT Hospital of Fudan University, Shanghai Key Laboratory of Visual Impairment and Restoration, Shanghai, 200031 China

**Keywords:** Macular telangiectasia type 1, Disorganization of the retinal inner layers, Optical coherence tomography angiography, Ellipsoid zone disruption

## Abstract

**Background:**

To evaluate the structural changes associated with visual acuity (VA) in patients with idiopathic macular telangiectasia (MT) type 1 using multimodal imaging modalities.

**Methods:**

A retrospective study of 14 patients with MT type 1 and of 10 eyes from 10 healthy individuals as age-matched controls was conducted. The medical records of patients who had undergone colour fundus photography, spectral domain optical coherence tomography (OCT), fluorescein angiography and OCT angiography were reviewed. Central macular thickness (CMT), the areas of macular oedema and ellipsoid zone (EZ) disruption, EZ length, disorganization of the retinal inner layers (DRIL) and external limiting membrane (ELM) disruption, as measured by spectral domain OCT; and vascular density and the foveal avascular zones (FAZ) of the superficial capillary plexus (SCP) and deep capillary plexus (DCP), as measured by OCT angiography, were assessed in MT type 1 eyes and correlated with VA.

**Results:**

The mean baseline best-corrected VA of MT type 1 eyes was 0.45 ± 0.28. The mean CMT was 385.19 ± 75.21 μm in MT type 1 eyes and 252.43 ± 15.77 μm in contralateral eyes (Z = − 4.113, *p <* 0.001). The mean vessel density of the DCP was lower in MT type 1 eyes (47.25 ± 4.69%) than in contralateral eyes (53.93 ± 2.94%) and normal eyes (59.37 ± 2.50%) (Z = − 3.492, − 4.099; *p <* 0.001, *<* 0.001). The baseline logMAR VA was correlated with CMT (*r* = 0.682, *p* = 0.007), SCP density (*r* = − 0.652, *p* = 0.012), DCP density (*r* = − 0.700, *p* = 0.005), total area of EZ disruption (*r* = 0.649, *p* = 0.012); and total lengths of EZ (*r* = 0.681, *p* = 0.007), ELM (*r* = 0.699, *p* = 0.005) and DRIL (*r* = 0.707, *p* = 0.005) disruption in the 1-mm-diameter foveal region in MT type 1 eyes.

**Conclusions:**

Decreased DCP density and the presence of DRIL may be predictive biomarkers of VA in MT type 1. CMT, SCP density, total area of EZ disruption, and lengths of EZ and ELM disruption within the 1-mm-diameter central region were strongly associated with VA.

## Background

Idiopathic macular telangiectasia (MT) is characterized by abnormally dilated and tortuous capillaries around the fovea for no known reason. MT was originally termed idiopathic juxtafoveolar retinal telangiectasis, and was classified by Gass and Oyakawa into three categories: exudation, non-exudation and combination with or without nervous system vasculopathy [1]. Based on recent advances in imaging that have led to better characterization of the disease, Yannuzzi et al. have recently defined two distinct forms of the disease, MT type 1 (aneurysmal telangiectasia) and MT type 2 (perifoveal telangiectasia) [2].

MT type 1 is typically a unilateral disease that is found more often in men and is clinically associated with loss of retinal transparency, salient extensive ectasia, lipid exudates, arteriolar aneurysms, deeply right-angled venules, and intra-retinal cystoid degeneration mainly on the temporal side of the macula [2]. Previous studies of patients with MT type 1 have assessed the correlations of visual acuity (VA) with photoreceptor inner segment–outer segment (IS/OS) disruption and the cystoid space using spectral domain optical coherence tomography (SD-OCT), and that with the microvascular density of the superficial capillary plexus (SCP) and deep capillary plexus (DCP) using OCT angiography (OCTA) [3, 4]. However, few studies have explored the associations of SD-OCT- and OCTA-derived parameters with VA.

Although SD-OCT can be effective for evaluating the thickness of vascularized layers by automatic segmentation [5, 6], OCTA provides more detailed information of the vascular networks. OCTA is a new technology used as an auxiliary examination to provide high-resolution imaging of retinal morphology and sensitive identification of the vascular layers [7], and has become a critical method for evaluating retinal vascular disorders such as diabetic retinopathy [8, 9], retinal vein occlusion [10, 11] and MT types 1 [4, 12, 13] and 2 [14, 15].

The aims of our study were to evaluate the OCTA and SD-OCT features of MT type 1, to compare SCP and DCP microvascular densities between normal and contralateral eyes, and to correlate VA with central macular thickness (CMT), areas of macular oedema and ellipsoid zone (EZ) disruption, lengths of the EZ, disorganization of the retinal inner layers (DRIL) and external limiting membrane (ELM) disruption, as measured by OCT; and with vascular density and the foveal avascular zone (FAZ) areas of the SCP and DCP, as measured by OCTA.

## Methods

This was a retrospective study that enrolled consecutive patients with MT type 1 diagnosed from December 2014 to September 2017 at the outpatient clinic of the Eye & ENT Hospital of Fudan University, Shanghai, China. This study was approved by the Institutional Review Board of the Eye and ENT Hospital of Fudan University. All procedures were performed according to the principles of the Declaration of Helsinki. Written informed consent was obtained from all participants or their guardians.

Patients underwent comprehensive ocular examinations in both eyes, including best-corrected VA (BCVA) evaluation, intraocular pressure reading, slit-lamp biomicroscopic ophthalmoscopic exam, dilated fundoscopy, fundus photography (Topcon TRC50LX; Topcon, Tokyo, Japan), OCTA (Optovue RTVue XR 100 Avanti, Fremont, CA, USA), SD-OCT (Heidelberg Engineering, Heidelberg, Germany) and fluorescein angiography (FA, Topcon TRC501X; Topcon). None of the patients in this study had undergone previous ocular treatment. As the control group, 10 right eyes imaged by SD-OCT and OCTA from 10 healthy individuals without any ocular diseases who were of similar age and sex as the MT type 1 cases were evaluated.

The clinical criteria used for diagnosing MT type 1 were confirmed by FA and included the following: (1) FA detection of unilateral, visible telangiectasis, aneurysms during the early stage and fluorescein leakage during the late stage of MT type 1, (2) OCTA detection of telangiectasis, aneurysms and decreased vascular densities of the SCP and DCP and (3) SD-OCT detection of intra-retinal oedema and hard exudates. Patients with neovascular maculopathies (e.g., age-related macular degeneration, polypoidal choroidal vasculopathy, retinal angiomatous proliferation, angioid streaks and other causes of secondary MT, including Coats’ disease, Leber disease, retinal vein occlusion and radiation retinopathy) were excluded. Also excluded were patients with diabetes, hypertension, ischemic heart disease, a history of vitreoretinal surgery, ophthalmic disorders excluding mild refractive errors and mild cataracts, or an oral history of anti-oestrogen tamoxifen for breast cancer.

SD-OCT was performed with 6-mm line scans (vertical and horizontal) across the centre of the fovea. A 19 line scans that covered a 20 × 15° (5.8 × 4.3 mm) area centred on the fovea was obtained in all eyes, with 20 automated real-time means per scan on high-resolution mode. The CMT in the 1-mm-diameter central region of the macula according to the Early Treatment of Diabetic Retinopathy Study thickness map was measured using Spectralis software. The ELM is formed by the junctions between the inner segments of photoreceptors and Müller cells [16]. The EZ is formed by the reflectivity generated from the high mitochondrial density of the outermost portion of the inner photoreceptor segments [17]. To calculate the area of EZ disruption, the intensity of the EZ band and border of the EZ disruption were measured using the plot profile function in ImageJ software (National Institutes of Health, Bethesda, MD, USA) and the grayscale image obtained by SD-OCT. The border of the disrupted EZ area was defined as a decrease in EZ reflectivity of 2 SD compared with an EZ band in the normal retina [18]. DRIL was defined as the inability to identify and differentiate any of the boundaries of the ganglion cell layer–inner plexiform layer (IPL) complex, inner nuclear layer, and outer plexiform layer (OPL) [19]. All images were obtained by well-trained operators (GJL, TWY). All data were measured within a 1-mm-diameter region encompassing the foveal centre and also on three B-scans performed immediately above and below.

Two independent observers (GJL, TWY) assessed the OCTA data using the best-quality 3 × 3-mm scan and controlled the corrected segmentation for the 14 patients before reporting the data. Vascular retinal layers were divided into the SCP, DCP, outer retina and choroidal layers by OCTA. The FAZ and density of the macula were assessed using flow density map software Angio-Analytics (Optovue RTVue XR 100 Avanti). The signal strength index of all images was greater than 60. Whole-image data were used to measure the microvascular densities in the SCP and DCP layers.

### Statistical analysis

BCVA values were converted into logarithm of the minimum angle of resolution values (logMAR) for statistical evaluation. Quantitative data (mean BCVA, CMT, vascular density, FAZ areas of the SCP and DCP) were compared among MT type 1, contralateral and normal eyes by the Mann–Whitney test and Wilcoxon test using IBM SPSS Statistics v19 (SPSS Inc., Chicago, IL, USA). Significance was defined as *P* < 0.05. Pearson’s correlation coefficient analyses were used to identify the factors associated with VA. The correlation was defined as none/very weak at *r* < 0.1, weak at 0.1 < *r* < 0.3, moderate at 0.3 < *r* < 0.6, strong at 0.6 < *r* < 0.8, or very strong at 0.8 < *r* < 1 [19].

## Results

**Fig. 1:**
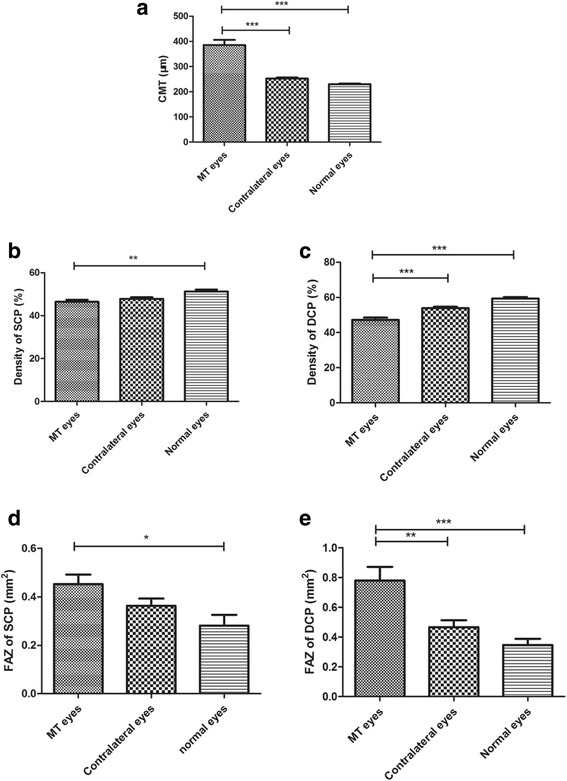
Comparisons among idiopathic macular telangiectasia (MT) type 1 eyes, contralateral eyes and normal eyes. (**a**) Central macular thickness (CMT). (**b**) Density of the superficial capillary plexus (SCP). (**c**) Density of the deep capillary plexus (DCP). (**d**) Foveal avascular zone (FAZ) area of the SCP. (**e**) FAZ area of the DCP. *p < 0.05, ** *p* < 0.01, *** *p* < 0.001

**Fig. 2:**
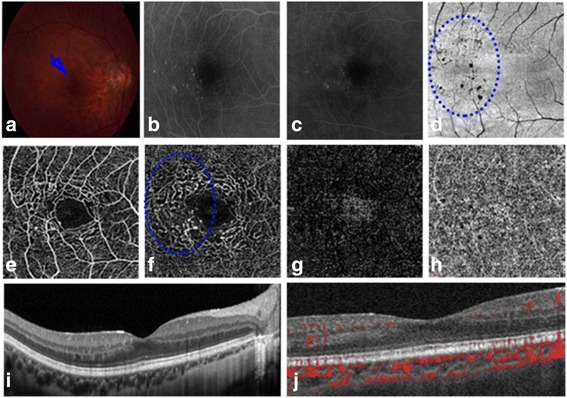
Imaging of the right eye of Patient 4. Capillary variations are visible from the inner to the outer plexiform layers on the temporal side of the macula. (**a**) Blue arrow shows sparse micro-aneurysms. (**b**) Fluorescein angiography (FA) reveals hyperfluorescence from micro-aneurysms on the temporal side of the macula during the early stage of MT type 1. (**c**) FA showing leakage of fluorescein due to vascular endothelial cell injury during the late stage of MT type 1. (**d**) No lesion is visible in the inner and outer segment junction (IS/OS) and blue-dotted oval shows sparse micro-aneurysms on en-face OCT. (**e**) OCT angiography (OCTA) reveals decreased capillary density in the superficial capillary plexus. (**f**) OCTA image showing the transformational foveal avascular zone (FAZ), aneurysms, and telangiectasia in the deep capillary plexus (DCP) on the temporal side of the macula. (**g** and **h**) OCTA images show no changes in the outer or choroidal layers, respectively. (**i**) SD-OCT shows areas of hyper-reflectance from the inner to the outer plexiform layers on the temporal side. (**j**) OCTA showing blood flow

A total of 14 eyes in 14 patients (8 men and 6 women) were included in the study. The mean ± SD age was 55.07 ± 12.74 years (range, 34–75 years). The BCVA values (mean ± SD) in the MT type 1, contralateral and control eyes at baseline (logMAR) were 0.45 ± 0.28 (range, 20/20 to 20/200), 0.07 ± 0.07 (range, 20/20 to 20/32) and 0.02 ± 0.04 (range, 20/20 to 20/25), respectively. The CMT values (mean ± SD) in the MT type 1, contralateral and normal eyes were 385.19 ± 75.21 (range, 224–487) μm, 252.43 ± 15.77 (range, 225–280) μm and 230.30 ± 7.04 (221–241) μm, respectively (Fig. 1). The fundus photography revealed oedematous maculae in 13 eyes, in which macular oedema appeared as hypo-reflective areas, and hard exudates and a few aneurysms appeared as hyper-reflective areas on SD-OCT at the first visit. In the remaining patient, SD-OCT displayed multiple disorganized hyper-reflective points from the IPL to the OPL (Fig. 2). The CMT values were higher in the 14 MT type 1 eyes than in the contralateral and normal eyes (Z = − 3.296, Z = − 4.101; *p* < 0.001, *p* < 0.001). An interrupted EZ structure was found in 13 patients. On FA, abnormally dilated and tortuous capillaries and aneurysms were observed during the early phase and intense hyperfluorescence and parafoveal leakage during the late phase, mainly in the temporal hemisphere. The demographic characteristics of the patients are listed in Table 1.

**Table 1:** Characteristics of idiopathic macular telangiectasia type 1 patients and normal patients

	MT eyes	Fellow eyes	Normal eyes
Number of eyes	14	14	10
Age (years)	55.07 ± 12.74 (34-75)	55.07 ± 12.74 (34-75)	48.80 ± 9.32(36-60)
Sex (men/women)	8/6	8/6	6/4
BCVA(LogMAR)	0.45 ± 0.28	0.07 ± 0.07	0.02 ± 0.04
BCVA (Snellen equivalent)	20/44 (20/20-20/200)	20/23 (20/20-20/32)	20/21 (20/20-20/25)
Microvascular density of SCP(%)	46.47 ± 3.42 (38.25-51.90)	47.78 ± 2.89 (42.52-52.33)	51.30 ± 2.81 (46.46-54.50)
Microvascular density of DCP(%)	47.25 ± 4.69 (39.90-54.60)	53.93 ± 2.94 (48.36-57.83)	59.37 ± 2.50 (54.62-61.96)
FAZ of SCP (mm^2^)	0.45 ± 0.15 (0.22-0.71)	0.36 ± 0.11 (0.21-0.59)	0.28 ± 0.14 (0.07-0.55)
FAZ of DCP (mm^2^)	0.78 ± 0.35 (0.25-1.45)	0.47 ± 0.17 (0.33-0.94)	0.35 ± 0.13 (0.11-0.57)
CMT (μm)	385.19 ± 75.21 (224-487)	252.43 ± 15.77 (225-280)	230.30 ± 7.04(221-241)
Mean areas with photoreceptor damage (mm^2^)	3.22 ± 2.02 (0-5.81)		

*BCVA* best-corrected visual acuity, *SCP* superficial plexus, *DCP* deep plexus, *FAZ* foveal avascular zone, *CMT* centre macular thickness, *MT* idiopathic macular telangiectasia

In the OCTA examination, decreased vessel density and telangiectasis in the SCP and DCP were observed in all of the MT type 1 eyes, and these observations were much clearer compared with those on FA or OCT when observing telangiectasis variations in real time. The high resolution of OCTA enabled the clearest view of the vascular structure of the retina. The mean vessel density value of the DCP (47.25 ± 4.69%) was lower in MT type 1 eyes than in contralateral eyes (53.93 ± 2.94%) and normal eyes (59.37 ± 2.50%) (Z = − 3.492, Z = − 4.099; *p <* 0.001, *p <* 0.001). The FAZ areas of the SCP and DCP were larger in MT type 1 eyes (0.45 ± 0.15 mm^2^ and 0.78 ± 0.35 mm^2^, respectively) than in normal eyes (0.28 ± 0.14 mm^2^ and 0.35 ± 0.13 mm^2^, respectively) (Z = − 2.372, Z = − 3.455; *p* = 0.018, *p* = 0.001; Fig. 1).

The baseline logMAR VA was correlated with the CMT (*r* = 0.682, *p* = 0.007), densities of the SCP (*r* = − 0.652, *p* = 0.012) and DCP (*r* = − 0.700, *p* = 0.005), total area of EZ disruption (*r* = 0.649, *p* = 0.012); and the lengths of EZ (*r* = 0.681, *p* = 0.007), ELM (*r* = 0.699, *p* = 0.005) and DRIL (*r* = 0.707, *p* = 0.005) disruption in the 1-mm-diameter foveal region in MT type 1 eyes. The associations of baseline SD-OCT and OCTA parameters with baseline logMAR VA are shown in Table 2.

**Table 2:** Associations of baseline spectral-domain optical coherence tomography and optical coherence tomography angiography parameters with baseline logMAR visual acuity

Parameters	MT type 1 eyes	*r*	*p* value
FAZ of SCP (mm^2^)	0.45 ± 0.15	0.451	0.106
FAZ of DCP (mm^2^)	0.78 ± 0.35	0.521	0.056
Microvascular density of SCP (%)	46.47 ± 3.42	−0.652	0.012
Microvascular density of DCP (%)	47.25 ± 4.69	− 0.700	0.005
CMT (μm)	385.19 ± 75.21	0.682	0.007
Total area of macular oedema (mm^2^)	0.08 ± 0.10	0.443	0.112
Largest area of macular edema (mm^2^)	0.08 ± 0.09	0.512	0.061
Length of EZ disruption (μm)	642.26 ± 349.66	0.681	0.007
Area of EZ disruption (mm^2^)	0.93 ± 1.22	0.27	0.351
Total area of EZ disruption (mm^2^)	3.22 ± 2.02	0.649	0.012
Length of ELM disruption (μm)	372.57 ± 279.16	0.699	0.005
Length of DRIL disruption (μm)	480.79 ± 340.73	0.707	0.005

*p*-values indicate the relationships between logMAR VA and spectral domain OCT and OCTA parameters

*OCT* spectral domain optical coherence tomography, *OCTA* optical coherence tomography angiography, *logMAR* logarithm of the minimal angle of resolution, *VA* visual acuity, *MT* idiopathic macular telangiectasia, *FAZ* foveal avascular zone, *SCP* superficial capillary plexus, *DCP* deep capillary plexus, *CMT* central macular thickness, *EZ* ellipsoid zone, *ELM* external limiting membrane, *DRIL* disorganization of retinal inner layers, *r* Pearson’s correlation coefficient

Early-stage MT type 1 was observed in Patient 4 (Fig. 2). In this patient, central macular oedema (CME) was not detected by SD-OCT (Fig. 2g), but the patient had blurred vision and abnormal microvascular variations in the IPL and OPL. Aneurysms were subsequently seen on FA (Fig. 2b) and OCTA. The telangiectasis of SCP and DCP was clearly revealed by OCTA (Fig. 2c). The vascular changes were barely evident in the SCP but prominent in the DCP.

In addition, the DCP density was decreased in MT type 1 eyes compared with contralateral and normal eyes. The FAZ areas in the SCP and DCP were larger in MT type 1 eyes than in contralateral and normal eyes.

## Discussion

Few studies have evaluated the correlations between anatomic factors and baseline VA in MT type 1 [3, 4]. Takayama et al. reported strong associations between intra-retinal cystoid spaces and IS/OS alterations and retinal sensitivity [3]. Matet et al. found that logMAR VA was inversely correlated with SCP and DCP densities [4]. The current study demonstrated strong correlations of baseline logMAR VA with DRIL and microvascular DCP density, and associations were also found between baseline logMAR VA and CMT, SCP density, lengths of EZ and ELM disruption within the 1-mm-diameter central region of the macula, and the total area of EZ disruption. Because these SD-OCT- and OCTA-derived anatomic parameters can provide further information on VA, they could be useful in determining therapeutic regimens and for developing patient counselling strategies.

In the present study, a strong correlation was found between baseline logMAR VA and DRIL. The inner retinal layers play crucial roles in neural transmission from the photoreceptors to the retinal ganglion cells. The inability to distinguish the retinal layer boundaries likely represents partial destruction of the inner retinal cells, thus interrupting transmission between the photoreceptors and the ganglion cells [20]. Sun et al. were the first to report that DRIL is correlated with worse VA in eyes with diabetic macular oedema [21]. All of the MT type 1 eyes in the present study had DRIL, which was positively correlated with baseline logMAR VA. Furthermore, the reduced viable tissue in DRIL may further decrease the demand for a blood supply, resulting in loss of the deep vascular plexus [22]. This view was confirmed by our findings that the DCP density was decreased in all patients with MT type 1 and was correlated with logMAR VA. The ELM, which exhibits several different junctional complexes between Muller and rod/cone photoreceptor cells, may be an indispensable factor for the survival of photoreceptor cells. The integrity of the ELM and EZ may be regarded as predictors of a better VA prognosis [10]. In our study, the lengths of EZ and ELM disruption within the 1-mm-diameter central region and the total area of EZ disruption were positively correlated with the baseline logMAR VA.

With the rapid development of imaging techniques, OCTA has become a convenient and noninvasive method for evaluating ocular variations in retinal vascular diseases (particularly variations in the deep capillary) that can be used at every visit to the clinic [23]. SD-OCT and FA enable gross evaluation of changes in the vascular network. Because of the limited visualization of FA due to dye leakage, we were unable to observe the deep vascular plexus in more detail using this method. However, OCTA has the particular advantage of visualizing real-time dynamic changes in various retinal layers, and enables gross quantification of capillary density in several retinal layers simultaneously [23]. Accordingly, we can use OCTA to assess the reductions in capillary density in both the SCP and DCP in comparison with those of the contralateral healthy eye, despite the lack of an accurate measuring technique. Moreover, the alterations in DCP density and FAZ area are specific parameters for analysis at follow-up. Two unique characteristics of MT type 1 eyes were revealed in the present patients by OCTA. First, there was a negative correlation between DCP density in the lesion and logMAR VA in our cases. Second, decreased capillary density variation in the deep layer was a predictor of very-early-stage MT type 1. Birol et al. confirmed in an animal model that the deep capillary bed is conducive to the oxygen requirements of the photoreceptor layer [24]. In their study, the primary oxygen supply to photoreceptor layers was derived from the choroidal circulation, but 10%–15% was derived from the retinal circulation. As oxygenation of the fovea is somewhat different from that of the perifoveal retina, it would be useful to discuss this and any other potentially relevant correlations regarding the foveal and perifoveal areas. We speculate that the vascular variation in the DCP and DRIL may predict hypoxidosis in the photoreceptor layer. Of the 13 patients in the study of Birol et al., 12 had characteristics of macular oedema rather than retinal atrophy. However, among our cases, patient 4, who had obviously distorted capillaries and aneurysms during the early stage, and leakage during the late stage according to FA, had slightly blurred vision without CME, but the DCP density and FAZ area already showed obvious transformations. One possible explanation for this finding is that the abnormal morphology and structure from the IPL to OPL on the temporal side of the macula are also related to telangiectasia. Gass and Oyakawa [1] suggested that disruption of the deeper capillary plexus predominantly causes variation in the retinal vasculature and that the late diffuse fluorescence is derived from the outer retina. It has been reported that in MT type 1, changes occur earlier in the deep capillary bed than in the superficial capillary bed [25], which was confirmed in the present series.

The limitations of our study included its retrospective design and the small sample size due to the low prevalence of MT type 1. The captured images inevitably have artefacts despite being obtained by two experienced observers (GJL and TWY).

## Conclusion

The greater the scale of EZ disruption and/or lengths of DRIL and ELM disruption in the lesion, the worse the vision. Decreased DCP density is potentially an earlier indicator of MT type 1 than are macular oedema and EZ layer disruption. We speculate that variation in structure from the IPL to the OPL on OCT and morphological changes on OCTA are manifestations of very-early-stage MT type 1.

## Abbreviations

BCVA: best-corrected visual acuity
CMT: central macular thickness
DCP: deep capillary plexus
DRIL: disorganization of the retinal inner layers
ELM: external limiting membrane
EZ: ellipsoid zone
FA: fluorescein angiography
FAZ: foveal avascular zone
MT type 1: idiopathic macular telangiectasia type 1
OCTA: optical coherence tomography angiography
SCP: superficial capillary plexus
SD-OCT: spectral-domain optical coherence tomography
VA: visual acuity
